# Histamine H1 receptor antagonists selectively kill cisplatin-resistant human cancer cells

**DOI:** 10.1038/s41598-021-81077-y

**Published:** 2021-01-15

**Authors:** Nobuki Matsumoto, Miku Ebihara, Shiori Oishi, Yuku Fujimoto, Tomoko Okada, Toru Imamura

**Affiliations:** 1 Cell Regulation Laboratory, Bionics Program, Tokyo University of Technology Graduate School of Bionics, Computer and Media Science, Hachioji, Japan; 2 School of Bioscience and Biotechnology, Tokyo University of Technology, Hachioji, Tokyo, Japan; 3 National Institute of Advanced Industrial Science and Technology (AIST), Tsukuba, Ibaraki Japan

**Keywords:** Cancer therapy, Cancer therapeutic resistance, Targeted therapies, Drug therapy, Mechanism of action

## Abstract

Cancer therapy is often hampered by the disease’s development of resistance to anticancer drugs. We previously showed that the autonomously upregulated product of fibroblast growth factor 13 gene (*FGF13*; also known as FGF homologous factor 2 (*FHF2*)) is responsible for the cisplatin resistance of HeLa cisR cells and that it is likely responsible for the poor prognosis of cervical cancer patients treated with cisplatin. Here we show that cloperastine and two other histamine H1 receptor antagonists selectively kill HeLa cisR cells at concentrations that little affect parental HeLa S cells. The sensitivity of HeLa cisR cells to cloperastine was abolished by knocking down *FGF13* expression. Cisplatin-resistant A549 cisR cells were similarly susceptible to cloperastine. H2, H3, and H4 receptor antagonists showed less or no cytotoxicity toward HeLa cisR or A549 cisR cells. These results indicate that histamine H1 receptor antagonists selectively kill cisplatin-resistant human cancer cells and suggest that this effect is exerted through a molecular mechanism involving autocrine histamine activity and high-level expression of *FGF13*. We think this represents a potential opportunity to utilize H1 receptor antagonists in combination with anticancer agents to treat cancers in which emergent drug-resistance is preventing effective treatment.

## Introduction

Cancer cells often develop resistance to the anticancer drugs used against them. To study the development of resistance at the molecular level and investigate the underlying mechanisms, we previously established HeLa cisR cells from HeLa S cells by culturing them in media containing incrementally increasing concentrations of cisplatin^1^. The resultant HeLa cisR cells exhibited resistance to cisplatin, other platinum anticancer drugs, and copper. The strongly upregulated expression of *FGF13* gene and protein in HeLa cisR cells was responsible for the platinum anticancer drug resistance, as evidenced by the disappearance of resistance when *FGF13* expression was suppressed^1^. Furthermore, in preoperative cervical cancer biopsy samples from patients with poor prognoses after cisplatin chemoradiotherapy, FGF13-positive cells were detected more abundantly than in the biopsy samples from patients with good prognoses^1^. Thus, FGF13 appears to be a promising target for platinum drug-resistant anticancer therapy, as well as a useful marker for selection of anticancer drugs to achieve better prognoses.

The mechanism by which FGF13 confers cisplatin resistance is poorly understood. We found that intracellular platinum concentrations were kept low in HeLa cisR cells. When FGF13 expression was suppressed, both the cells’ resistance to platinum drugs and their ability to keep intracellular platinum levels low were abolished. Overexpression of FGF13 in parental HeLa S cells led to greater resistance to cisplatin and reductions in the intracellular platinum concentration, but the effects were weak^1^. Moreover, these cisplatin-resistant cells also showed resistance to copper, suggesting the activity of a copper transporter, such as CTR1, which is also thought to be responsible for cisplatin incorporation, is suppressed by FGF13. Genes encoding SLC7A11 and SLC3A2, which together form a cystine/glutamate exchange transporter, are also greatly upregulated in HeLa cisR cells, suggesting biosynthesis of glutathione is upregulated in these cells. Subsequent confirmation that GST levels are modestly upregulated in the cells suggest a higher level of antioxidant activity^1^.

Since our initial publication of the findings summarized above, numerous studies have reported on FGF13′s actions to mitigate various cellular stresses in cancer cells. One of those reports proposed that FGF13 may serve as an enabler, allowing cancer cells to evade proteostatic stress triggered by oncogene activation^2^. Another report identified FGF13 as a target of CD271 in chemoresistant cells and showed that knocking down CD271 decreased *FGF13* expression and partially restored sensitivity to fotemustine, an anticancer drug^3^. *FGF13* expression was also reported to correlate with the aggressively metastatic nature of triple-negative breast cancer^4^.

Our aims in the present study were to identify compounds able to exert cytotoxic effects on HeLa cisR cells, making them potentially useful anticancer agents, and to better understand the mechanisms by which FGF13 confers cisplatin resistance.

## Materials and methods

### Chemical compounds and vehicles

Cisplatin and histamine dihydrochloride were obtained from Wako Chemicals (Osaka, Japan); cloperastine hydrochloride was from Tokyo Chemical Industry Co., Ltd. (TCI); clemastine fumarate was from Funakoshi Co., Ltd. (Tokyo, Japan); desloratadine was from TCI; nizatidine, pitolisant, and JNJ-7777120 were from Cayman Chemical (Michigan, USA). To prepare concentrated stock solutions, cisplatin was dissolved in water containing 0.9% NaCl to a concentration of 500 µg/ml; cloperastine was dissolved in water; clemastine fumarate, desloratadine, nizatidine, and JNJ-7777120 were dissolved in ethanol. Every experiment on the effect of a drug was conducted with the solvent alone as a control.

### Cell culture

Parental HeLa S cells (a derivative of the HeLa cell line kindly gifted by Dr. Handa at the Tokyo Institute of Technology) were maintained in Eagle’s minimum essential medium (Eagle’s MEM: Nissui Pharmaceutical Co., Ltd., Tokyo, Japan) supplemented with 10% fetal bovine serum (FBS: Wako Chemicals), 2 mM L-glutamine (Wako Chemicals), 1 mM sodium pyruvate (Wako Chemicals), 0.075% NaHCO_3_ (Wako Chemicals), and 20 mM HEPES (Dojindo Laboratories, Kumamoto, Japan) at 37℃ under 5% CO_2_. HeLa cisR cells, a cisplatin-resistant HeLa S cell derivative, were maintained in HeLa S cell growth medium supplemented with 6 µg/ml cisplatin to maintain their drug resistance. HeLa cisR cells were somewhat smaller than HeLa S cells when suspended, resulting in smaller Frontal Scatter in flow cytometric analyses. By contrast, adherent HeLa S cells yielded larger numbers of cells than HeLa cisR cells when the cells reached confluency. HeLa cisR cells were confirmed to have originated from HeLa S cells with short tandem repeat (STR) analysis (performed by Takara Bio Inc., Osaka, Japan). The STR profile of HeLa S cells was also confirmed to be similar to that of HeLa cells (ATCC CCL-2) and HeLa S3 cells (ATCC CCL-2.2).

### Screening approved drugs that exert cytotoxicity toward HeLa cisR cells

All drugs (1494 chemical compounds) approved by the Pharmaceuticals and Medical Devices Agency, Japan (PMDA) were provided for screening by the Platform Project for Supporting Drug Discovery and Life Science Research (Basis for Supporting Innovative Drug Discovery and Life Science Research (BINDS)) from Japan Agency for Medical Research and Development (AMED). The screening system is similar to the assessment of cytotoxicity based on cell proliferation, as described below, except that each compound was applied to the surface of culture well bottom (0.1 µL/well) before the test cells were seeded.

### Establishment of cisplatin-resistant A549 cisR cells

Cisplatin-resistant A549 cisR cells were established as previously described for HeLa cisR cells. Briefly, parental human lung carcinoma A549 cells (RCB0098; freshly obtained from RIKEN Bio Resource Center) were maintained in growth medium composed of Dulbecco's modified eagle medium (DMEM; Wako Chemicals) with 10% FBS and 60 µg/ml kanamycin (Meiji Seika Pharma, Tokyo, Japan). By subculturing these cells in the presence of incrementally increasing concentrations of cisplatin, we established a highly resistant subline, A549 cisR cells, which were maintained in growth medium containing 3 µg/ml cisplatin.

### Measurement of cytotoxicity based on cell proliferation

Cytotoxicity was analyzed as described previously^1^. Cells (5 × 10^3^ cells/100 µL/well) were seeded onto 96-well plates and incubated at 37 °C under 5% CO_2_. After 24 h, various compounds were added concomitantly in a volume of 2 µL/well to achieve the desired final concentrations, after which the cells were incubated for an additional 3 days. Cytotoxicity was measured based on cell numbers as follows. Cell Counting Kit (CCK)-8 reagent (a modified MTT assay system; Dojindo Laboratories, 5 µL/well) was added to each well, and the plates were incubated for 5 h, after which the absorbance at 450 nm was measured using a microplate reader. The ratios of the cell numbers in the drug-containing cultures to those in the control drug-free cultures were then calculated. To evaluate the cytotoxic effects exerted by two compounds, the compounds were added to the cultures concomitantly without delay.

### Evaluation of cytotoxicity toward mixed cell populations

The cytotoxicity of various compounds was tested with HeLa S cells, HeLa cisR cells, and mixed populations of the two. For the separate cultures, 4 × 10^3^ HeLa S cells/well or 1.5 × 10^4^ HeLa cisR cells/well were analyzed. For the mixed population (combined culture), HeLa S cells (2 × 10^3^ cells/well) and HeLa cisR cells (7.5 × 10^3^ cells/well) were mixed and then seeded into 96-well plates. After incubation for 1 day to allow the cells to attach, selected compounds were added in 2 µL/well to achieve the desired final concentrations, and the cells were incubated for an additional 3 days. Cytotoxicity was then measured based on cell numbers as described above.

### Measurement of histamine-induced cell proliferation

Cells (5 × 10^3^ cells/100 µL/well) were seeded into 96-well plates and incubated at 37 °C under 5% CO_2_ for 1 day. The attached cells were washed twice with serum-free medium and then serum-starved by incubation for 3 days in culture medium containing 0.1% serum. Thereafter, the cells were washed with serum free medium incubated for 7 days in 100 µL/well of culture medium containing selected concentrations of FBS and histamine (added in 2 µL/well). Cell numbers were then measured as described above, and the ratios of the cell numbers in histamine-containing cultures to those in the control histamine-free cultures were calculated.

### Evaluation of the effects of histamine and cloperastine on cell proliferation

Cells (5 × 10^3^ cells/100 µL/well) were seeded into 96-well plates and incubated at 37 °C under 5% CO_2_. After 1 day, histamine was added in 2 µL/well, and the cells were incubated for an additional day. Cloperastine was then added in 2 µL/well, and the cells were incubated for another 2 days. During the final 5 h of incubation, cell numbers and ratios were determined as described above.

### RNA isolation and quantitative RT-PCR

Total RNA was isolated from cells using a FastGene RNA Basic Kit (Nippon Genetics, Tokyo, Japan). The total RNA (250 ng) was then reverse-transcribed to cDNA using ReverTra Ace qPCR RT Master Mix (TOYOBO, Osaka, Japan) with a random primer (9 mer). The specific primer sets used in the quantitative RT-PCR were as follows: for *β-actin*, 5′-TCCCTGGAGAAGAGCT-3′ (forward) and 5′-GTTGGCGTACAGGTCT-3′ (reverse); for *FGF13* (all variants), 5′-ACAAGCCTGCAGCTCATTTT-3′ (forward) and 5′-CTTTTGCCCTCACTGGCTAC-3′ (reverse); for *HRH1*, 5′-AGGTCCCTCCCTTCCTTCTC-3′ (forward) and 5′-CACCACCAGCATCTTTTGGC-3′ (reverse). The quantitative analyses were performed with Quant Studio5 (Thermo Fisher Scientific, Tokyo, Japan) using these primers and Thunderbird SYBR qPCR Mix (TOYOBO, Osaka, Japan) following the manufacturers’ instructions.

### Flow cytometric analyses

Flow cytometric analyses were conducted using a Model SH-800 flow cytometer (SONY, Tokyo, Japan) following the manufacturer's instructions. For flow cytometric analysis of the cell cycle, growing cells in 100-mm dishes were incubated for 3 days in their respective growth media (control) or in medium containing 1 µg/ml cisplatin or 100 µM cloperastine. The cells were then washed, fixed with 70% ethanol, stained with propidium iodide (PI) and subjected to flow cytometry. For flow cytometric analysis of apoptosis/cell death, growing cells in 100-mm dishes were incubated for 1 day with selected compounds and washed. The unfixed cells were then stained with Annexin V-FITC and PI using an ApoAlert Annexin V-FITC Apoptosis Kit (Clonetech Laboratories, Tokyo, Japan), after which the intensities of the FITC and PI fluorescences were analyzed using a flow cytometer and plotted.

### Statistical analyses

**Figure 1:**
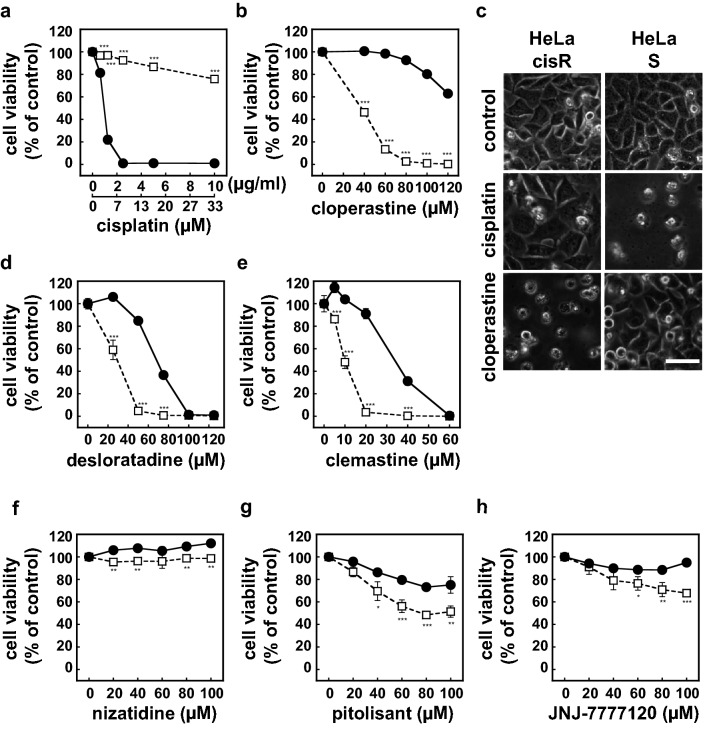
Cloperastine and other H1 receptor antagonists selectively kill HeLa cisR cells. (**a**,**b**,**d**–**h**) HeLa cisR cells (open squares) and HeLa S cells (filled circles) were cultured for 3 days with or without the indicated concentrations of cisplatin (**a**), the histamine H1 receptor antagonists cloperastine (**b**), desloratadine (**d**), or clemastine (**e**), or the H2 receptor antagonist nizatidine (**f**), H3 receptor antagonist pitolisant (**g**), or H4 receptor antagonist JNJ-7777120 (**h**). Cell viability during the final 5 h of the culture period was evaluated using WST-8 colorimetric assays. Open squares, HeLa cisR cells; filled circles, HeLa S cells. The results are presented as means ± S.D. of sextuplicate (n = 6) (**a**,**b**,**d**,**e**) or quadruplicate (n = 4) (**f**–**h**) samples. Four separate experiments yielded essentially the same results. Note that only H1 receptor antagonists resulted in 0% cell viability. Also note that error bars are not visible in many places due to their small size. The significance of the difference between the results from HeLa S and HeLa cisR cells at the same concentration of the indicated compound was determined with ANOVA (***p < 0.001; **p < 0.01; *p < 0.05). (**c**) HeLa cisR and HeLa S cells cultured for 3 days with 10 µg/ml cisplatin or 100 µM cloperastine were photographed under a phase contrast microscope. Bar, 50 µm.

Comparisons of the results expressed a percentages of control (e.g., HeLa cisR cells vs. HeLa S cells at particular cisplatin concentrations in Fig. 1) actually means comparison among raw data from samples with two factors and two levels. We therefore used ANOVA for the statistical analysis of these data. Dunnett's post hoc test was used to verify the significance because the analyses described above always compare the no-drug (zero concentration) measurements with measurements at a particular drug concentration. The significance of difference(s) are shown as * for p < 0.05, ** for p < 0.01, or *** for p < 0.001 upon verification with a post hoc test.

## Results

### Identification of cloperastine and other H1 receptor antagonists as selective and strong cytocidal drugs for HeLa cisR cells

HeLa cisR cells show greater resistance to cisplatin than the parental HeLa S cells (Fig. 1a,c). However, by screening nearly the entire approved drug library (1492 drugs currently approved by PMDA, Japan), we found that cloperastine, an antitussive drug with H1 histamine receptor antagonist activity^5^, killed 50% of HeLa cisR cells at a concentration of 40 µM and 100% at 80 µM (Fig. 1b,c). Cloperastine was much less cytotoxic toward cisplatin-sensitive HeLa S cells, with IC_50_ higher than 120 µM. Even at 80 µM, 90% of HeLa S cells remained viable in the presence of cloperastine (Fig. 1b). Two other H1 receptor antagonists, desloratadine and clemastine, exhibited similar cytotoxicity toward HeLa cisR cells, though desloratadine was somewhat less selective. Desloratadine and clemastine killed nearly 100% of HeLa cisR cells at 50 µM and 20 µM, respectively (Fig. 1d,e). By contrast, the H2 receptor antagonist nizatidine showed little cytotoxicity toward either HeLa cisR or HeLa S cells (Fig. 1f), and while the H3 receptor antagonist pitolisant and H4 receptor antagonist JNJ-7777120 were both selectively cytotoxic toward HeLa cisR cells, killing was incomplete (55% and 70% viable, respectively) at the highest achievable concentrations tested (100 µM; Fig. 1g,h).

**Figure 2:**
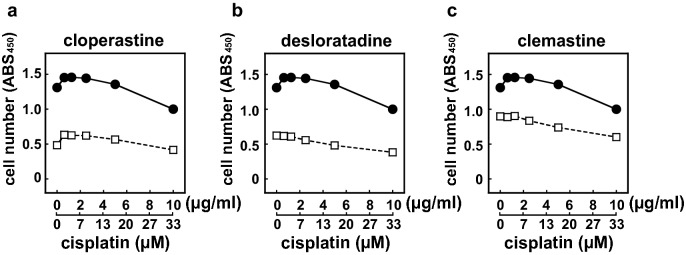
Cisplatin cytotoxicity toward HeLa cisR cells is not synergistically enhanced by H1 receptor antagonists. HeLa cisR cells were cultured for 3 days in the absence (closed circles) or presence (open square) of 40 µM cloperastine (**a**), 30 µM desloratadine (**b**), or 10 µM clemastine (**c**) with the indicated concentrations of cisplatin. Cell numbers during the final 5 h of the culture were evaluated using WST-8 colorimetric assays. The results are presented as means ± S.D. of quintuplicate (n = 5) samples. Three separate experiments yielded essentially the same results.

**Figure 3:**
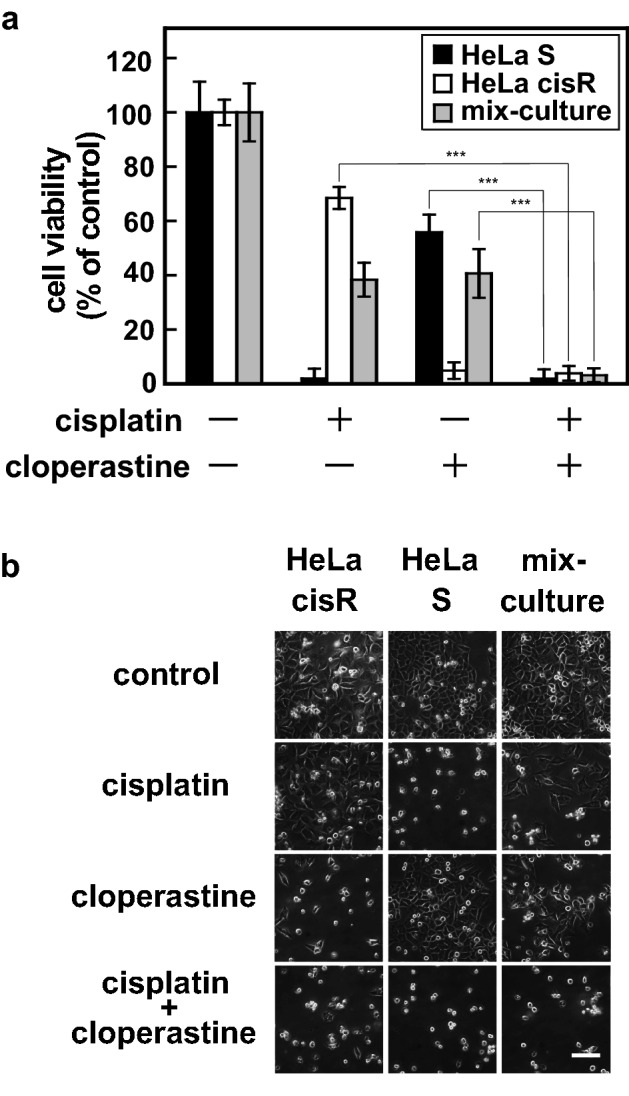
Combined use of cloperastine and cisplatin effectively kill both cisplatin-resistant and cisplatin-sensitive cancer cells in a mixed population. (**a**) Cytotoxicities of cloperastine and/or cisplatin toward HeLa S cells and HeLa cisR cells were analyzed separately or together as a mixed population. For the separate cultures, 4 × 10^3^ HeLa S cells/well, or 1.5 × 10^4^ HeLa cisR cells/well were analyzed. For the mixed population (combined culture), HeLa S cells (2 × 10^3^ cells/well) and HeLa cisR cells (7.5 × 10^3^ cells/well) were mixed and seeded into each well of a 96-well plate. After incubation for 1 day to allow the cells to attach, 100 µM cloperastine and/or 10 µg/ml (33 µM) cisplatin were added, and the cells were incubated for an additional 3 days, after which cell viability was measured. The results are presented as means ± S.D. of sextuplicate samples. Four separate experiments yielded essentially the same results. The significance of the difference between the selected pairs of results was determined with ANOVA (***p < 0.001). (**b**) Cells treated as in (**a**) were photographed under a phase contrast microscope. Bar, 100 µm.

HeLa cisR cells and HeLa S cells were treated without or with H1 receptor antagonists at their approximate IC_50_, and the effect of cisplatin was evaluated to see if there is any synergism between these drugs. We found that cisplatin cytotoxicity toward HeLa cisR cells is not synergistically enhanced by cloperastine, desloratadine, or clemastine (Fig. 2a–c). However, when an H1 receptor antagonist combined with cisplatin was applied to a mixed culture of HeLa S and HeLa cisR cells, the combination of cloperastine and cisplatin killed all the cells in the mixed cell population (Fig. 3a,b).

### Cytotoxicity of H1 receptor antagonists positively correlates with resistance to cisplatin and FGF13 expression

**Figure 4:**
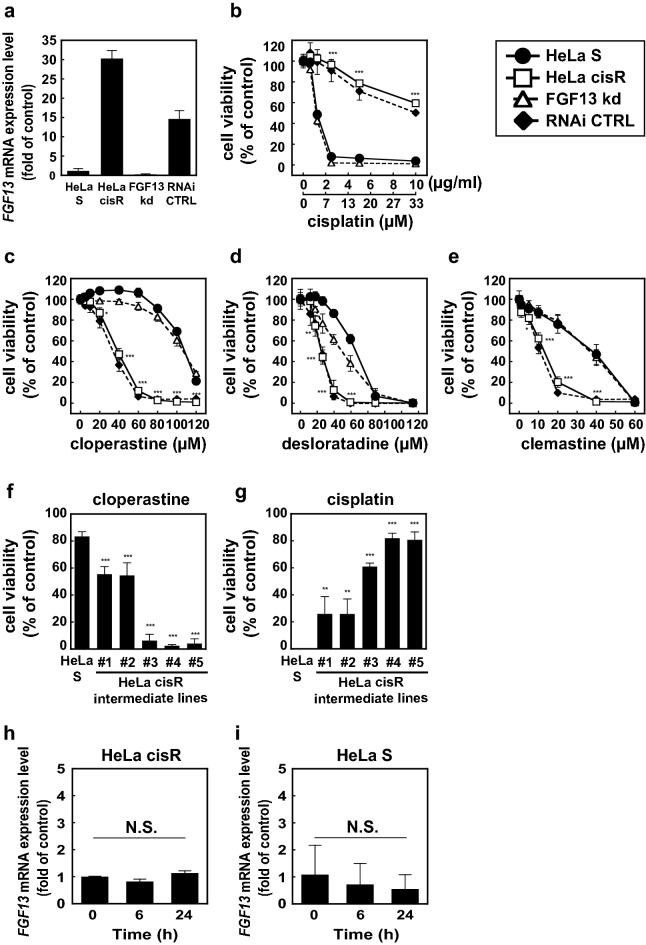
Cytotoxicity of H1 receptor antagonists is dependent on the levels of FGF13 expression and cisplatin resistance. (**a**) Relative expression level of *FGF13* in each cell line. (**b**–**e**) Cytotoxicities of cisplatin (**b**) and H1 receptor antagonists cloperastine (**c**), desloratadine (**d**), and clemastine (**e**) toward HeLa S cells, HeLa cisR cells, HeLa cisR cells in which *FGF13* expression was suppressed by targeted siRNA expression (FGF13kd), and HeLa cisR cells in which degenerate siRNA was expressed (RNAi CTRL). Open squares, HeLa cisR cells; filled circles, HeLa S cells; open triangles, FGF13kd cells; filled diamonds, RNAi CTRL cells. (**f**) Cytotoxicity of 100 µM cloperastine toward HeLa S cells and five intermediate HeLa cisR cell lines, which were collected during the process of establishing the HeLa cisR cells (#1 to #5, adapted to 0.42, 0.84, 2.0, 6.0 and 8.0 µg/ml cisplatin in this order) and which differ in their expression of *FGF13* mRNA. (**g**) Cytotoxicity of 10 µg/ml (33 µM) cisplatin toward HeLa S cells and the five intermediate HeLa cisR cell lines. (**h**,**i**) Separate cultures of HeLa cisR cells (**h**) and HeLa S cells (**i**) were treated with cloperastine for the indicated periods, after which relative expression of *FGF13* mRNA was quantitated using RT-qPCRs. The results are presented as mean ± S.D. of sextuplicate (**b**–**e**) or triplicate (**a**,**f**–**i**) samples. Three separate experiments yielded essentially the same results. In panels (**b**–**g**) the significance of the difference between the results from HeLa S and HeLa cisR cells was determined with ANOVA (***p < 0.001; **p < 0.01; *p < 0.05).

Interestingly, an *FGF13* knock-down line of HeLa cisR cells (FGF13kd, Fig. 4a), which are cisplatin-sensitive (Fig. 4b), survived in the presence of cloperastine at concentrations cytotoxic to HeLa cisR cells, in which *FGF13* is upregulated (Fig. 4a,c). Desloratadine (Fig. 4d) and clemastine (Fig. 4e) also exhibited FGF13-dependent cytotoxicity toward HeLa cisR cells. Similarly, RNAi control HeLa cisR cells (RNAi CTRL), in which high levels of *FGF13* expression were retained, are killed by the H1 receptor antagonists, just like HeLa cisR cells (Fig. 4c–e). Five intermediate cell lines collected during the process of establishing the HeLa cisR cells (#1 to #5, adapted to 0.42, 0.84, 2.0, 6.0 and 8.0 µg/ml cisplatin) differed in their levels of *FGF13* expression^1^ and showed corresponding differential sensitivity to cloperastine, which increased with increases in *FGF13* expression and decreases in cisplatin sensitivity (Fig. 4f,g)^1^. On the other hand, cloperastine did not significantly affect *FGF13* expression in either HeLa cisR cells (Fig. 4h) or HeLa S cells (Fig. 4i).

### H1 receptor expression is upregulated in HeLa cisR cells, and histamine enhances HeLa cisR cell proliferation

**Figure 5:**
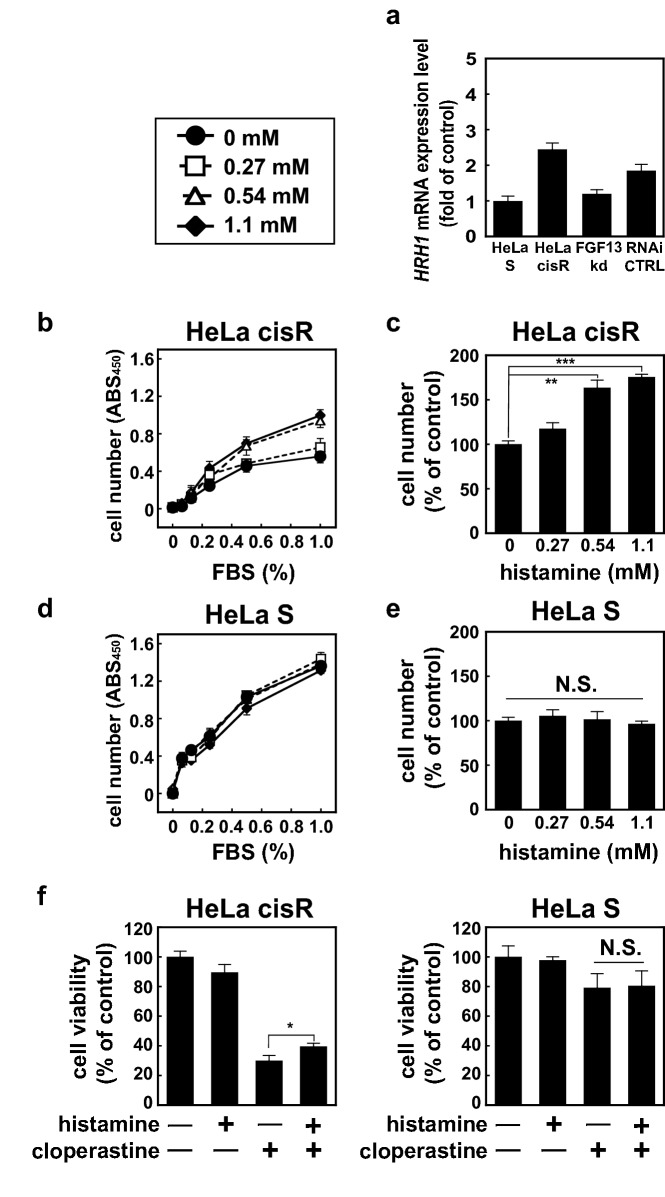
Expression of histamine H1 receptors is upregulated in HeLa cisR cells, and histamine enhances HeLa cisR cell proliferation. (**a**) Relative expression of histamine H1 receptor mRNA in HeLa S cells, HeLa cisR cells, FGF13kd cells, and HeLa cisR RNAi CTRL cells. (**b**,**d**) Effects of histamine on cell proliferation were examined in the culture media containing the indicated concentrations of FBS. Filled circles, 0 µg/ml; open squares, 50 µg/ml; open triangles, 100 µg/ml; filled diamonds, 200 µg/ml. (**c**,**e**) The data collected in the 1% FBS-containing medium in (**b**,**d**) are plotted to help comparison. (**f**) Effects of 500 µg/ml (2.7 mM) histamine on the viability of HeLa cisR and HeLa S cells in the absence or presence of 120 µM cloperastine. In this experiment, attached cells in the 10% FBS-containing medium were first treated with histamine for 1 day and then incubated in the absence or presence of cloperastine for 2 days, after which cell viability was measured as described in the methods. The results are presented as means ± S.D. of triplicate (**a**), quadruplicate (**b**–**d**), or sextuplicate (**f**) samples. Three separate experiments yielded essentially the same results. The significance of the difference between the selected pairs of results was determined with ANOVA (***p < 0.001; **p < 0.01; *p < 0.05; N.S., not significant).

Given that histamine H1 receptor antagonists selectively kill HeLa cisR cells in an FGF13-dependent manner, we examined H1 receptor expression in HeLa cisR cells. We found that H1 receptor mRNA expression is upregulated in HeLa cisR cells as compared to HeLa S cells, though not in FGF13kd cells (Fig. 5a). Moreover, histamine dose-dependently enhanced proliferation of HeLa cisR cells, with the greatest enhancement achieved at 1.1 mM (200 µg/ml) (Fig. 5b,c), but did not affect HeLa S cell proliferation (Fig. 5d,e). Consistent with that activity, histamine at 2.7 mM (500 µg/ml) improved the viability of HeLa cisR cells but not HeLa S cells in the presence of cloperastine (Fig. 5f).

Regarding the concentrations of histamine used, according to one study^6^, the pathophysiological concentrations of histamine in circulating blood may vary from 40 ng/mL in a healthy individual to 121 ng/ml in an ischemic heart disease patient. Another study, focusing on the effects of histamine on mast cell degranulation^7^, used histamine at concentrations ranging from 0.3 mM (33 µg/mL) to 32 mM (3556 µg/mL). Thus, the precise concentration of histamine in the cellular microenvironment may be much higher than that in the circulating blood. In the present study, therefore, to identify the potential involvement of histamine in the regulation of cancer cells, we experimentally determined the concentrations of histamine to be used.

### Effects of cisplatin and cloperastine on cell cycling and apoptosis in HeLa cisR cells

**Figure 6:**
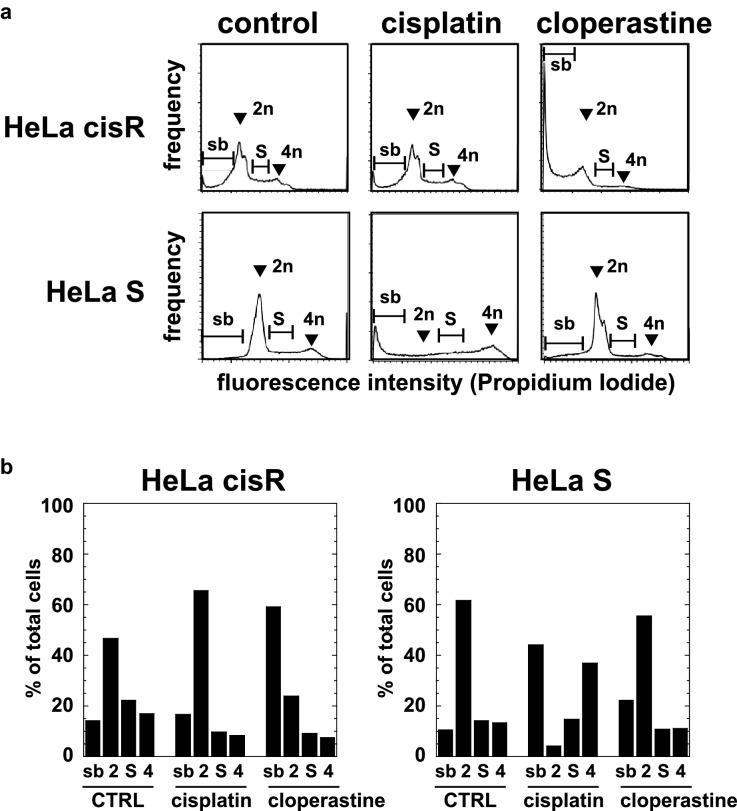
Cloperastine increases the HeLa cisR cell fraction in sub G1 phase. (**a**) Flow cytometric analysis of the cell cycle. Growing HeLa cisR and HeLa S cells were cultured for 3 days in their respective growth media (control) or in medium containing 1 µg/ml cisplatin or 100 µM cloperastine. The cells were then fixed, the cellular DNA stained with propidium iodide (PI), and the fluorescence intensity and frequency analyzed using a flow cytometer and plotted. Arrowheads and brackets indicate sub G1 phase (**sb**), G1 phase (**2n**), S phase (**S**), and G2/M phase (**4n**) for each cell line and treatment. Two separate experiments yielded essentially the same results. (**b**) Relative cell numbers in each cell cycle phase in panel a were quantified using ImageJ software and plotted as percentages of the total cells. The labels indicate sub G1 phase (**sb**), G1 phase (**2**), S phase (**S**), and G2/M phase (**4**) for each cell line and treatment.

The effects of cisplatin and cloperastine on HeLa cisR and HeLa S cell cycling were examined using flow cytometry. The cells were cultured in FBS-containing growth medium in the presence of a compound for 3 days prior to fixation and staining with PI for flow cytometric analysis. Serum-containing medium was used because the cell cycles of HeLa cisR and HeLa S cells could not be synchronized, even in the absence of serum (Matsumoto, unpublished observation). Among control HeLa S cells, G1 phase cells (2n) predominated (Fig. 6a,b). When cells were treated with 1 µg/ml cisplatin, there was a dramatic decrease in cells in G1 phase and increases in cells in sub-G1 phase (sb) and G2/M phase (4n) (Fig. 6a,b). Treatment with 100 µM cloperastine weakly increased numbers of sub-G1 and G2/M phase cells but did not significantly reduce G1 phase cells (Fig. 6a,b). In contrast to HeLa S cells, a relatively small fraction of control HeLa cisR cells were in G1 phase (Fig. 6a,b). When cells were treated with 1 µg/ml cisplatin, the cell fractions at each cell cycle phase were largely unchanged (Fig. 6a,b). Treating HeLa cisR cells with 100 µM cloperastine dramatically increased the sub-G1 phase fraction and reduced the S phase and G2/M phase fractions (Fig. 6a,b), suggesting the occurrence of apoptosis.

**Figure 7:**
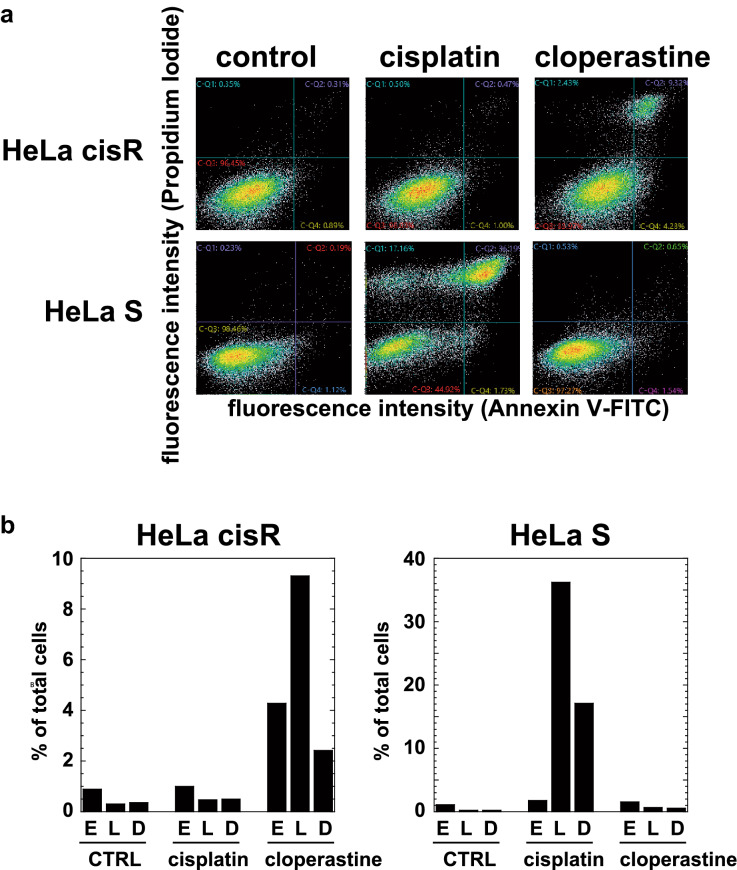
Cloperastine increases the HeLa cisR cell fraction undergoing apoptosis/cell death. (**a**) Flow cytometric analysis of cell death. Growing HeLa cisR and HeLa S cells were cultured for 24 h in their respective growth media (control) or in medium containing 1 µg/ml cisplatin or 100 µM cloperastine. The cells were stained with Annexin V-FITC and PI, and the intensities of the FITC and PI fluorescences were analyzed using a flow cytometer and plotted. In each panel, the bottom left section represents Annexin V^LOW^PI^LOW^ (live) cells, the bottom right section Annexin V^HIGH^PI^LOW^ cells (early phase apoptosis), the top right section Annexin V^HIGH^PI^HIGH^ cells (late phase apoptosis), and the top left section Annexin V^LOW^PI^HIGH^ cells (plasma membrane disintegration). (**b**) Relative cell numbers in each phase of apoptosis/cell death in panel a were quantified using ImageJ software and plotted as percentages of the total cells. The labels indicate early phase apoptosis (**E**), late phase apoptosis (**L**), and plasma membrane disintegration (**D**) for each cell line and treatment.

We then used Annexin V-PI staining assays to examine the effects of cisplatin and cloperastine on apoptosis/cell death among HeLa cisR and HeLa S cells. For these assays, growing cells in heterogenous cell-cycle phases were cultured with cisplatin or cloperastine for 1 day before the unfixed cells were stained with Annexin V and PI prior to cytometric analysis. Cells undergoing early phase apoptosis are shown as Annexin V^HIGH^PI^LOW^ cells, while those undergoing late phase apoptosis are shown as Annexin V^HIGH^PI^HIGH^ cells. Cells undergoing plasma membrane disintegration are shown as Annexin V^LOW^PI^HIGH^ cells. The majority of control HeLa cisR and HeLa S cells were Annexin V^LOW^PI^LOW^, which indicates that there had been little artificial damage to the analyzed cells (Fig. 7a,b). When HeLa cisR cells were treated with cloperastine, the incidences of both early phase and late phase apoptosis increased markedly (Fig. 7a,b). When HeLa cisR cells were treated with 1 µg/ml cisplatin, the cell distribution did not change significantly as compared to control cells (Fig. 7b). By contrast, when HeLa S cells were treated with 1 µg/ml cisplatin, there were dramatic increases in cells in the late phase of apoptosis or exhibiting plasma membrane disintegration (Fig. 7a,b). Treating HeLa S cells with 100 µM cloperastine did not greatly affect the numbers of cells falling into either of those groups (Fig. 7a,b).

### Signal transduction pathways downstream of FGF receptor tyrosine kinases are not involved in the cloperastine cytotoxicity toward HeLa cisR cells

**Figure 8:**
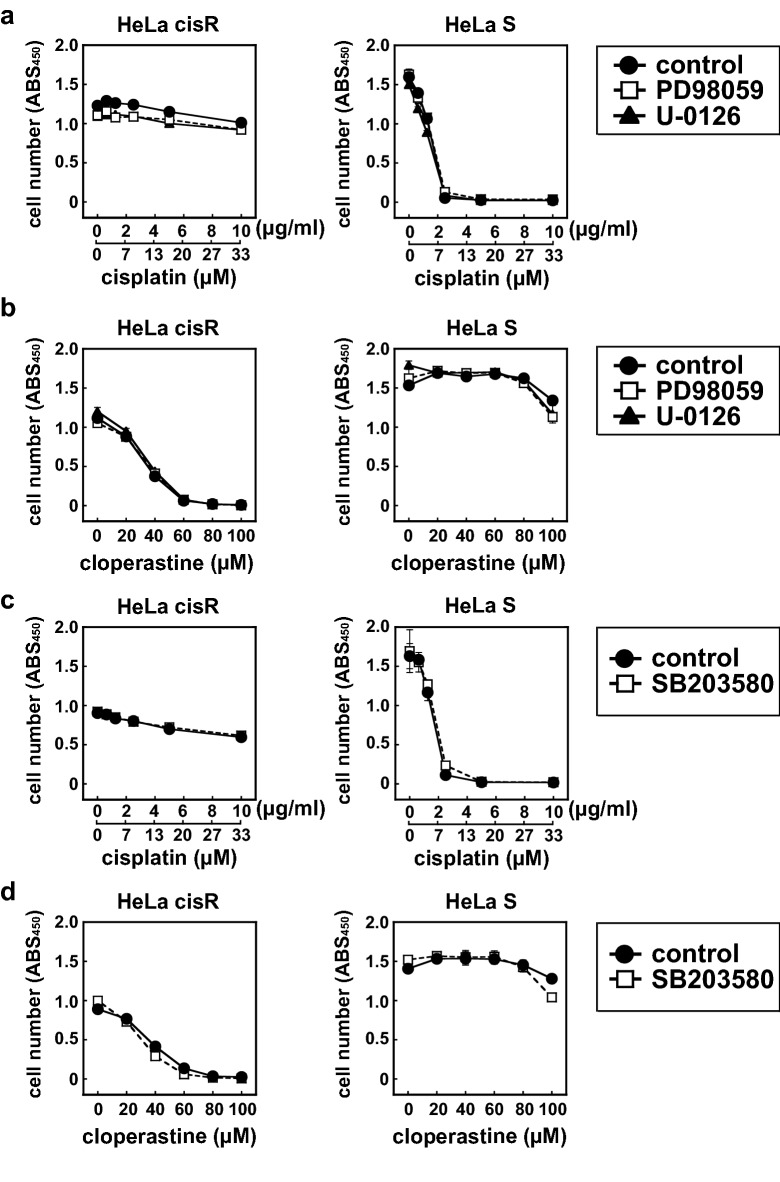
MEK and p38 MAPK signal transduction pathways are not involved in the cloperastine cytotoxicity toward HeLa cisR cells. (**a**,**b**) Effects of inhibitors of MEK on the cytotoxicity of cisplatin (**a**) and cloperastine (**b**) toward HeLa cisR and HeLa S cells. Filled circles, control; open squares, PD98059; filled triangles, U-0126. **c** and **d**, Effects of an inhibitor of p38 MAPK on the cytotoxicity of cisplatin (**c**) and cloperastine (**d**). Filled circles, control; open squares, SB203580. The results are presented as means ± S.D. of triplicate (a and b) or quadruplicate (c and d) samples. Three separate experiments yielded essentially the same results.

FGF13 reportedly exerts its effects through direct binding to multiple intracellular binding partners, but not FGF receptor tyrosine kinases^8^. For instance, FGF13 enhances p38 MAP kinase (MAPK) signaling by binding to its scaffold protein, Id2. To determine whether MAPK signaling is involved in FGF13′s apparent activity enhancing the cloperastine sensitivity of HeLa cisR cells, we examined the effects of several inhibitors of MAPK activation. PD98059 and U-0126 inhibit MAPK extracellular signaling-regulated kinase kinase (MEK), which is an intermediate in FGF receptor tyrosine kinase signaling, while SB203580 inhibits p38 MAPK signaling. As shown in Fig. 8, the cytotoxic effects of cisplatin (Fig. 8a,c) and cloperastine (Fig. 8b,d) toward HeLa S and HeLa cisR cells were not significantly affected by inhibition of MEK or p38 MAPK (Fig. 8a–d).

### Calcium channel blocker verapamil weakly enhances the cytotoxicity of H1 receptor antagonists, and the effect is not selective to HeLa cisR cells

**Figure 9:**
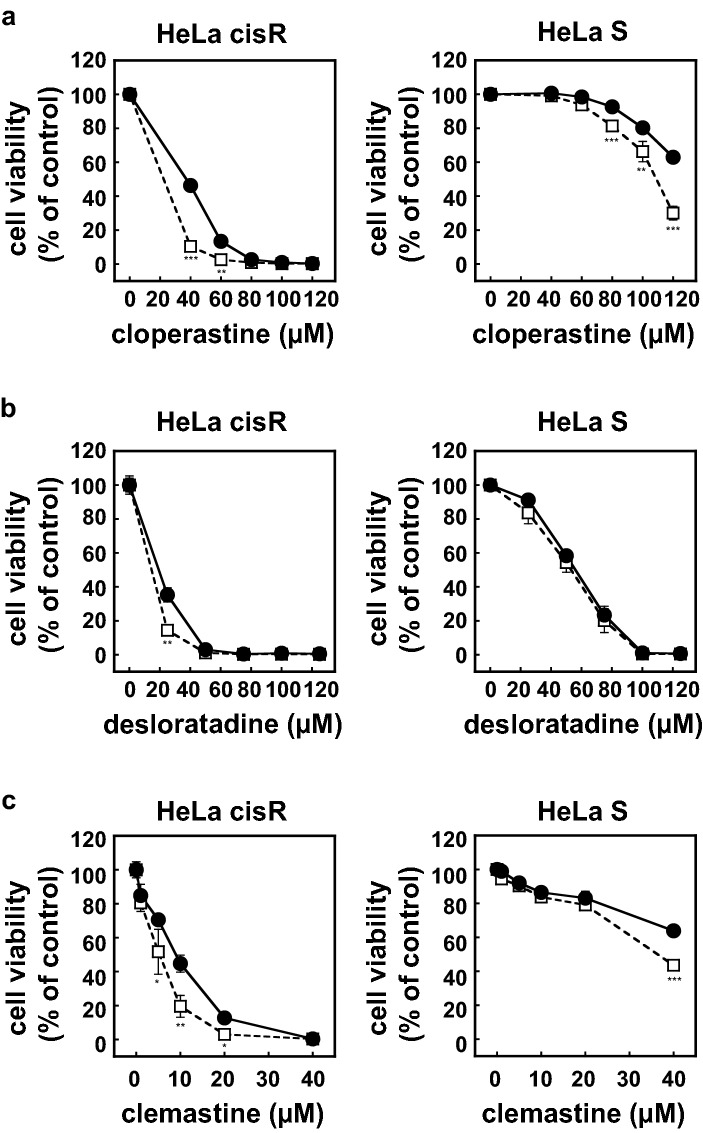
Weak enhancement of the cytotoxicity of H1 receptor antagonists by verapamil is not selective for HeLa cisR or HeLa S cells. HeLa cisR and HeLa S cells were cultured for 3 days with cloperastine (**a**), desloratadine (**b**), or clemastine (**c**) in the absence (filled circles) or presence (open squares) of 50 µM verapamil. Cell viability during the final 5 h of the culture was evaluated in WST-8 colorimetric assays. The results are presented as means ± S.D. of quadruplicate samples. Three separate experiments yielded essentially the same results. The significance of the difference between the results from control cells and verapamil-treated cells at the same concentration of the indicated compound was determined with ANOVA (***p < 0.001; **p < 0.01; *p < 0.05).

The stem-like properties of tumor side population cells are reportedly modulated by the calcium channel blocker verapamil, which enhances the cytotoxic effects of chemotherapeutic drugs and suppresses multidrug resistance by targeting the transport function of the P-glycoprotein^9^. We found that verapamil weakly enhanced the cytotoxicity of cloperastine toward both HeLa cisR and HeLa S cells (Fig. 9a) and had similar weak effects on the cytotoxicity of the other H1 receptor antagonists tested, desloratadine and clemastine (Fig. 9b,c). Moreover, these effects of verapamil were not selective for either HeLa cisR or HeLa S cells (Fig. 9a–c).

### Like HeLa cisR cells, cisplatin-resistant A549 cisR cells are selectively killed by H1 receptor antagonists

**Figure 10:**
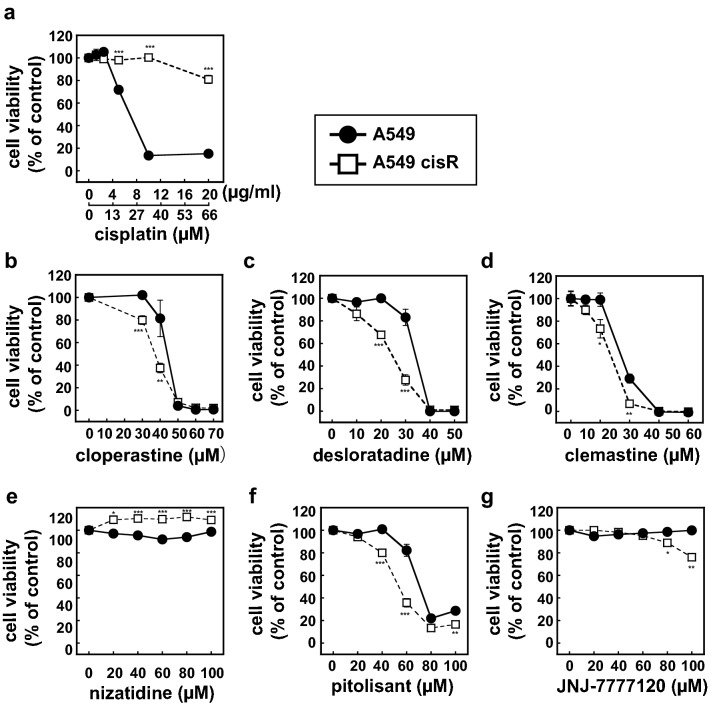
Cisplatin-resistant A549 cisR cells are selectively killed by histamine H1 receptor antagonists. A549 cisR cells were established from parental A549 lung cancer cells and subcultured in the presence of 3 µg/ml cisplatin. A549 cisR cells (open squares) and A549 cells (filled circles) were cultured for 3 days with the indicated concentrations of cisplatin (**a**); the histamine H1 receptor antagonist cloperastine (**b**), desloratadine (**c**), or clemastine (**d**); the H2 receptor antagonist nizatidine (**e**); H3 receptor antagonist pitolisant (**f**); or H4 receptor antagonist JNJ-7777120 (**g**). Cell viability was then assessed during the final 5 h of the culture using WST-8 colorimetric assays. The results are presented as means ± S.D. of sextuplicate (**a**–**d**) or quadruplicate (**e**–**g**) samples. Four separate experiments yielded essentially the same results. Note that only H1 receptor antagonists resulted in 0% cell viability. The significance of the difference between the results from A549 and A549 cisR cells at the same concentration of the indicated compound was determined with ANOVA (***p < 0.001; **p < 0.01; *p < 0.05).

Following the same experimental protocol used to establish HeLa cisR cells, we established cisplatin-resistant A549 cisR cells from parental A549 lung cancer cells (Fig. 10a). The A549 cisR cells were subcultured in the presence of 3 µg/ml cisplatin. As with HeLa cisR and HeLa S cells, cloperastine killed A549 cisR cells at lower concentrations than were needed to kill A549 cells (Fig. 10b), though the difference in sensitivity to cloperastine between A549 cisR and A549 cells was smaller than between HeLa cisR cells and HeLa S cells. Similar effects were seen with desloratadine and clemastine (Fig. 10c,d). Importantly, nearly 100% of the parental A549 cells were killed by 50 µM cloperastine, 40 µM desloratadine, or 40 µM clemastine (Fig. 10b–d), which is in contrast with > 120 µM, 100 µM, or 60 µM for the parental HeLa S cells, respectively (Fig. 1b,d,e). The H2 receptor antagonist nizatidine showed little cytotoxicity toward A549 cisR or A549 cells (Fig. 10e), and both the H3 receptor antagonist pitolisant and the H4 receptor antagonist JNJ-7777120 were selectively but less strongly cytotoxic toward A549 cisR cells (Fig. 10f,g).

## Discussion

### Histamine H1 receptor antagonists selectively kill cisplatin-resistant human cancer cells

Both cervical cancer-derived HeLa cisR cells and lung carcinoma-derived A549 cisR cells have been established as cells with autonomously acquired cisplatin resistance. We showed that these cells are preferentially killed by cloperastine and other histamine H1 receptor antagonists. The cloperastine sensitivity of HeLa cisR cells is dependent on endogenous expression of FGF13, the same molecule upon which cisplatin resistance depends. Thus, by treating with cisplatin and cloperastine in combination, a mixed population of HeLa cisR and parental HeLa S cells were effectively killed; cloperastine killed the HeLa cisR cells while cisplatin killed the HeLa S cells. This strongly suggests that combined use of cisplatin and cloperastine could potentially provide an effective means of killing human cancers composed of both cisplatin-sensitive and naturally-emerging cisplatin-resistant cells. Of course, as we previously proposed^1^, suppressing expression of *FGF13* using *FGF13* siRNA in cisplatin-resistant cells, if achievable, would be a straightforward way to treat FGF13-dependent cisplatin-resistant cells with cisplatin. In that case, our results predict that cloperastine would become less effective against the resultant FGF13kd cells.

### Practical utility of this finding

Interestingly, cloperastine is currently used as an approved antitussive drug to treat various types of cough, including the severe cough experienced by lung cancer patients. The regular clinical dose of cloperastine as a cough suppressant (10–20 mg three times daily for adults) is calculated to be approximately 3 µM. However, chronic toxicity tests performed in rats treated for 3 months with cloperastine hydrochloride at 15 mg/kg (45 µM) or 45 mg/kg (135 µM), and in dogs treated for 3 months with cloperastine hydrochloride at 20 mg/kg (60 µM) revealed no particular symptomatology or changes in hematochemical, hematological, or urinary parameters as compared to control animals and baseline values^10^. Thus, given the priority of cancer therapy, application of cloperastine to kill cisplatin-resistant cancer cells would be well within the scope of clinical testing. The two other H1 receptor antagonists tested in this study, desloratadine and clemastine, are also promising compounds with which to kill cisplatin-resistant cancer cells.

We showed previously and confirmed here that the cisplatin resistance of HeLa cisR cells is mediated by upregulation of *FGF13* expression^1^. We therefore suggest that detection of upregulated *FGF13* expression in biopsy samples could be utilized as an important biomarker, as we previously reported^1^. If high-level expression of FGF13 is detected in a cancer, then use of H1 receptor antagonists may be an effective approach to therapy.

Our results also indicate that clemastine suppresses the viability of both HeLa cisR and HeLa S cells at lower concentrations than desloratadine or cloperastine (Fig. 4c–e). However, as one of the aims of this study was to understand the molecular mechanism underlying cisplatin resistance, we chose to study cloperastine, which selectively exerts a strong cytotoxic effect on HeLa cisR cells but a weaker effect on cisplatin-sensitive HeLa S cells (Fig. 4c; LD_50_ of cloperastine was 40 µM for HeLa cisR cells and > 120 µM for HeLa S cells). A second reason for using cloperastine in this study was that earlier reports had established that the concentrations required to kill HeLa cisR cells may be safely used in patients (described above). In the future, however, desloratadine or clemastine may also prove useful once their safety has been established.

### Possible mechanism of histamine-dependent cell proliferation

Histamine reportedly stimulates proliferation of some types of cancer cells^11^. Indeed, we found that histamine dose-dependently enhanced the proliferation and viability of HeLa cisR cells, but not HeLa S cells, even in the presence of cloperastine (Fig. 5b–f). These results, together with the finding that H1 receptor antagonists reduce the viability of HeLa cisR cells, strongly suggest that histamine or basal signaling of the histamine receptor acts as an autocrine stimulator of HeLa cisR cell proliferation. HeLa cisR cells express histidine decarboxylase (data not shown), the key enzyme in the synthesis of histamine, though its significant enhancement as compared to HeLa S cells has not been confirmed.

It is thus likely that there is an autocrine loop between histamine production and histamine recognition by H1 receptors that supports HeLa cisR cell proliferation. However, in the reported case of colorectal cancer cells, histamine increased cell proliferation via H2/H4 receptors^11^. In the present study, by contrast, it appears to be the H1 receptor that mediates transduction of intracellular signals to induce/maintain HeLa cisR and A549 cisR cell proliferation in the presence of cisplatin. In addition, we found that parental A549 cells are also susceptible to H1 receptor antagonist-induced cytotoxicity, though less so than A549 cisR cells. Thus, the importance of histamine and the histamine receptor signaling pathway for survival and proliferation may vary depending on the cancer cell type. Indeed, the authors of a recent review concluded that histamine regulates cancer-associated biological processes during cancer development in multiple cell types, and the outcome will depend on the tumor cell type, the level of histamine receptor expression, and the associated signal transduction pathways^12^. Although those authors suggested that H4 receptors may be the most promising therapeutic target for cancer treatment^12^, our results show that the H4 receptor antagonist JNJ7777120 does not effectively decrease the viability of HeLa S, HeLa cisR, A549, or A549 cisR cells. Moreover, we observed that in addition to H1 receptor antagonists, the H3 receptor antagonist pitolisant decreased the viability of A549 cisR and A549 cells (Fig. 9). We suggest that the cytocidal efficacy of H1 receptor antagonists and other histamine receptor antagonists toward various cancers should be evaluated.

### What are the links among FGF13 activity, cisplatin resistance, and histamine signaling that enhance cell proliferation?

The actions of FGF13 include binding to and modulating the function of the voltage-gated sodium channel (VGSC)^8^; binding to Id2, a MAP kinase scaffold protein, to augment p38-modulated signaling^13^; and binding to microtubules to stabilize their structure^14^. The involvement of one or more of these activities in mediating HeLa cisR cells’ susceptibility to H1 receptor antagonists is not yet clear. Nonetheless, the positive correlation between the level of *FGF13* expression and susceptibility to cloperastine was clearly demonstrated in HeLa cisR cells, their intermediate cisplatin-resistant cells, and their parental HeLa S cells (Fig. 4c,f). Taken together, these findings strongly suggest that there is a molecular link between FGF13 activity, cisplatin resistance, and histamine signaling to enhance cell proliferation. Our findings that cloperastine does not affect *FGF13* expression and that FGF13 knock-down abolishes HeLa cisR cell sensitivity to cloperastine strongly suggest that FGF13 is situated upstream of the cloperastine sensitivity mechanism, and that the reverse is not the case. It was recently reported that Apigenin, a major plant flavone, inhibits histamine-induced cervical cancer tumor growth in vivo^15^. The authors of the study reported that histamine stimulates cervical tumor growth in vivo and in vitro by altering estrogen receptor (ER; ER-α and ER-β) expression levels and signaling. It was speculated that apigenin inhibited cervical tumor growth by reversing this histamine-induced abnormal ER signaling^15^. If this mechanism applies to HeLa cisR cells, then their growth in the presence of cisplatin may be more dependent on histamine-mediated abnormal ER signaling than is the growth of HeLa S cells. Although no direct linkage between FGF13 and ER signaling has yet been reported, it was recently found that FGF13 promotes metastasis of triple‐negative breast cancer, which tests negative for ERs^4^. This suggests FGF13 may provide a highly metastatic nature to these cancer cells that is not positively dependent on signaling by ERs. A fuller understanding of the mechanism by which FGF13 supports the activity of histamine receptor antagonists to effectively kill HeLa cisR cells and A549 cells will require further investigation.

### Additional implications of the present findings

It was recently reported that *FGF13* is expressed in cancer cells and that its high level contributes to the cells’ invasive^16^ and metastatic^4^ activity. We also found that HeLa cisR cells expressing high levels of FGF13 show greater mobility than HeLa S cells in in vitro scratch wound healing assays (data not shown). Together with our finding of a positive correlation between high *FGF13* expression and cloperastine sensitivity, we anticipate that highly metastatic cancer cells expressing high levels of FGF13 can be selectively killed with cloperastine and other H1 receptor antagonists, providing a new approach to treating metastatic cancers. This possibility will need to be examined in future studies.
